# Antenatal and Postnatal Sequelae of Oxidative Stress in Preterm Infants: A Narrative Review Targeting Pathophysiological Mechanisms

**DOI:** 10.3390/antiox12020422

**Published:** 2023-02-09

**Authors:** Silvia Martini, Arianna Aceti, Anna Nunzia Della Gatta, Isadora Beghetti, Concetta Marsico, Gianluigi Pilu, Luigi Corvaglia

**Affiliations:** 1 Neonatal Intensive Care Unit, IRCCS AOU S. Orsola, 40138 Bologna, Italy; 2 Department of Medical and Surgical Sciences, University of Bologna, 40138 Bologna, Italy; 3 Obstetrics Unit, Department of Obstetrics and Gynecology, IRCCS AOU S. Orsola, 40138 Bologna, Italy

**Keywords:** oxidative stress, pre-eclampsia, gestational diabetes, preterm birth, chorioamnionitis, white matter injury, bronchopulmonary dysplasia, retinopathy of prematurity, necrotizing enterocolitis, sepsis

## Abstract

The detrimental effects of oxidative stress (OS) can start as early as after conception. A growing body of evidence has shown the pivotal role of OS in the development of several pathological conditions during the neonatal period, which have been therefore defined as OS-related neonatal diseases. Due to the physiological immaturity of their antioxidant defenses and to the enhanced antenatal and postnatal exposure to free radicals, preterm infants are particularly susceptible to oxidative damage, and several pathophysiological cascades involved in the development of prematurity-related complications are tightly related to OS. This narrative review aims to provide a detailed overview of the OS-related pathophysiological mechanisms that contribute to the main OS-related diseases during pregnancy and in the early postnatal period in the preterm population. Particularly, focus has been placed on pregnancy disorders typically associated with iatrogenic or spontaneous preterm birth, such as intrauterine growth restriction, pre-eclampsia, gestational diabetes, chorioamnionitis, and on specific postnatal complications for which the role of OS has been largely ascertained (e.g., respiratory distress, bronchopulmonary dysplasia, retinopathy of prematurity, periventricular leukomalacia, necrotizing enterocolitis, neonatal sepsis). Knowledge of the underlying pathophysiological mechanisms may increase awareness on potential strategies aimed at preventing the development of these conditions or at reducing the ensuing clinical burden.

## 1. Introduction

Over the past decades, a growing body of evidence has shed light on the detrimental consequences of oxidative stress (OS) during antenatal and early postnatal life, leading to the definition of the so-called OS-related diseases in neonates [1].

Oxygen (O_2_) is key to the processes of mitochondrial oxidative phosphorylation. Under physiological conditions, more than 90% of O_2_ is reduced to water by cytochrome oxidase in the electron transport chain (ETC), while the remaining is reduced incompletely and contributes to the formation of reactive oxygen species (ROS), characterized by oxidizing and reducing properties. Superoxide anion (•O_2_^−^) is the most common biological ROS and results from O_2_ reduction with one electron. O_2_ reduction with two electrons forms hydrogen peroxide (H_2_O_2_) which is far more reactive than molecular oxygen [2]. Hydroxyl radical (•OH) is a powerful oxidant uncharged with one unpaired, but extremely reactive electron. Other ROS include ozone (O_3_), singlet oxygen (1 O_2_) and organic peroxides (ROOH). By reducing H_2_O_2_ through the Fenton reaction, transitional metals, such as reactive iron species, also contribute to ROS generation. Moreover, the reaction between •O_2_^−^ and nitric oxide radical (•NO) is key to the formation of reactive nitrogen species (RNS), which further contribute to the oxidative burden by forming additional ROS [3].

The biological mechanisms through which free radicals can be generated are various and include mitochondrial dysfunction, hypoxia, hyperoxia, ischemia-reperfusion, neutrophil and macrophage activation, endothelial cellular damage. ROS and RNS levels are tightly regulated by the concerted activity of several antioxidant enzymes (e.g., catalase (CAT), superoxide dismutase (SOD) and glutathione peroxidase (GP)) and of nonenzymatic components with antioxidant properties, such as flavonoids, bilirubin, uric acid, melatonin, transferrin, ferritin, lactoferrin, ceruloplasmin, albumin, vitamin C, vitamin E, carotenoids, acetylcysteine, exogenous selenium, zinc, magnesium and copper [1]. Reduced glutathione also contributes to control ROS via direct interaction or serving as a cofactor for ROS-detoxifying enzymes and, as such, is considered the largest antioxidative reservoir [4].

At physiological concentrations, ROS and RNS are involved in several biological functions, including immune responses, vascular regulation, mitogenic processes. However, when the redox homeostasis is shifted towards a reduction in antioxidant defenses or towards a free radical overproduction which cannot be counteracted by antioxidant systems, OS develops. OS is responsible for irreversible modifications of cell structures (e.g., lipid peroxidation, protein carbonylation, DNA oxidation), which alter cellular functions and are involved in several pathophysiological phenomena starting from pregnancy and continuing throughout postnatal life [5].

At birth, a rapid transition from the low-oxygen intrauterine environment to the oxygen-rich extrauterine environment occurs [6]. To front this transition, the fetal antioxidant systems are progressively upregulated during the last trimester of pregnancy: nonenzymatic antioxidants cross the placenta at increasing concentrations and the activity of endogenous antioxidant enzymes, such as SOD, more than doubles [6]. Moreover, data from autopsy on fetal brains have also demonstrated a developmental mismatch in the expression of antioxidant enzymes, with SOD expression lagging behind that of GP and CAT up to near-term gestational age (GA) [7]. This developmental upregulation of antioxidant systems, however, is disrupted in case of premature birth; hence, preterm neonates are particularly susceptible to OS because they are unable to effectively adapt their antioxidant defenses in response to the oxidative burden resulting from the transition to extrauterine environment, from the high FiO_2_ concentrations that can be required during neonatal resuscitation or to manage respiratory distress, and from their increased concentration of free iron [6,8]. The high metabolic turnover and the increased susceptibility to infections of preterm infants, secondary to their relative immunodeficiency, further contribute to ROS and RNS overproduction. Moreover, preterm birth often follows pregnancies complicated by such disorders as preeclampsia, fetal growth restriction and maternal infections, which are known to be associated with an increased oxidative burden. The exposure to the noxious effects of OS can therefore begin long before birth, influencing both pregnancy outcomes and neonatal health [5].

This narrative review aims to provide a detailed overview of OS-related pathophysiological mechanisms that contribute to the main diseases during pregnancy and in the early postnatal period following preterm birth. OS biomarkers and antioxidant treatments will not be discussed as recently addressed in targeted reviews [9,10,11,12].

## 2. OS and Pregnancy Disorders

**Figure 1:**
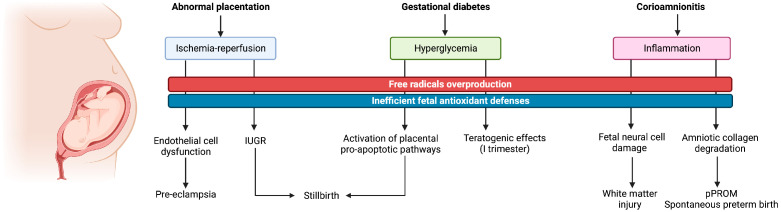
Graphical illustration of the main oxidative pathophysiological pathways related to pregnancy disorders and ensuing maternal and fetal consequences.

Several studies have proved the pivotal role that OS plays in the development of pregnancy related disorders, including early pregnancy loss, placental-induced hypertension, intrauterine growth restriction (IUGR), gestational diabetes and stillbirth [13]. An overview of the related pathophysiological pathways activated by OS and of the ensuing clinical consequences is illustrated in Figure 1.

In the first half of pregnancy, spiral arteries, the terminal branches of uterine arteries that supply blood to the endometrium, physiologically change their structure by replacing the myocytes of the internal lamina with fibrinoid, thus allowing to increase blood flow towards the placenta. Around 6–7 days after fertilization, the blastocyst keeps invading the endometrium, differentiating into cytotrophoblast (or extravillous trophoblast—the inner layer) and syncytiotrophoblast (the outer layer). All these changes are part of the progressive invasion of the extravillous trophoblast into the uterine stroma made of uterine glands, becoming firstly interstitial trophoblast and later, after the invasion of the endometrial vessels, endovascular trophoblast [14].

The intrauterine environment is fundamental for the implantation process: in vitro evidence has shown that the syncytiotrophoblastic layer of the early placenta is extremely sensitive to rising oxygen tension, and undergoes selective degeneration with this increase [15]. During human pregnancy, the fetoplacental unit is exposed to a progressively increasing gradient of oxygen concentrations between maternal tissues and fetal cells: the partial pressure of oxygen ranges between 20 mmHg in the first trimester and 55 mmHg in the second half of pregnancy, when the placenta metabolism becomes aerobic [16]. All these events are associated with the generation of free radicals, whose production increases with the rise in oxygen concentration. The syncytiotrophoblast is particularly sensitive to OS, firstly because it has a low expression of antioxidant enzymes, and secondly because the villous surface is directly exposed to the increasing oxygen concentration. Thus, if maternal antioxidant defenses are depleted or free radical production turns into OS, the ensuing damage to proteins, lipids and DNA may leads to a progressive and irreversible degeneration of the syncytiotrophoblast and to early pregnancy loss [17]. Consistently, immediately before abortion, a significant rise in biochemical markers of ROS-induced damage, such as lipid peroxidation products, has been observed [18].

Up to 2–3% of women in reproductive age experience recurrent pregnancy loss, defined as 3 or more consecutive abortions before 20 weeks of gestation; in about 50–60% of cases, there is no apparent causative factor, hence they are classified as idiopathic [19]. However, as the evidence of increased lipoperoxides and decreased antioxidant molecules in the plasma of patients with recurrent pregnancy loss suggests, OS in the endometrial environment may play a key role in these cases [20].

Even if the blastocyst is able to survive, however, OS determines an incomplete development of the spiral arteries with inadequate trophoblastic invasion, causing a progressive increase in placental vascular resistance which is responsible for reduced placental perfusion and for the ensuing ischemia-reperfusion injury [21]. A direct fetal consequence of this OS-related pathophysiological mechanism is placental-related IUGR, defined as an estimated fetal weight below the 10th percentile for GA, representing one of the leading cause of neonatal and perinatal mortality and morbidity [22]. Biri et al. demonstrated that, in singleton pregnancies diagnosed with IUGR, the concentration of lipid peroxidation products and of xanthine oxidase, an enzyme involved in ROS generation, was increased in placental tissue, maternal blood and cord blood samples compared to uncomplicated controls, whereas the levels of such scavenger enzymes as GP, CAT and SOD were increased in maternal serum and decreased in cord blood samples [21]. On the maternal side, growing evidence supports the detrimental role of OS in the etiopathogenesis of preeclampsia (PE). PE is a multisystem disorder that complicates 5–10% of human pregnancies, representing a major cause of maternal morbidity and being directly linked to IUGR and stillbirth. PE usually develops in the third trimester of pregnancy, when a rapid placental growth occurs [23]. In the presence of abnormal placentation and of ischemia-reperfusion placental injury, the maternal capability to counteract OS is further limited by the increased ROS production by the growing placenta, leading to the appearance of clinical symptoms of PE such as increased blood pressure and proteinuria, which indicate an endothelial dysfunction. Of note, by affecting inflammatory mediators, the rise in placental and serum levels of lipid peroxidation products associated with abnormal vascular placentation and with the ensuing OS may be involved in the endothelial cell dysfunction that is characterizes PE [24,25]. If the timing of delivery is not appropriately planned, a multisystem failure may occur; hence, PE is often associated with iatrogenic preterm birth. Typically, PE symptoms disappear shortly after the delivery of placenta [23].

Gestational diabetes mellitus (GDM) is defined as a glucose intolerance detected at any time during pregnancy [26]. In addition to abnormal placentation, the hyperglycemic environment ensuing from GDM is also responsible for OS generation [27]. Several studies have demonstrated an association between GDM and higher markers of OS in the placental tissue as well as in the maternal plasma and in the cord blood [28,29,30]. This association was observed even before the biochemical detection of increased glycated hemoglobin (HbA1c) [28]. Moreover, a direct correlation between HbA1c levels, which indicate a poor glycemic control, and lipid peroxidation end products, such as malondialdehyde, has also been documented in maternal serum [29]. Interestingly, in vitro studies on diabetic rat models have shown that hyperglycemia-induced OS causes increased concentrations of pro-apoptotic proteins, such as Bcl2, in the placental tissue [31]. From a clinical perspective, OS in GDM may have GA-dependent implications. In particular, when GDM is already present since the first trimester of pregnancy, it may increase the offspring susceptibility to such malformations as cardiac or neural tube defects [32,33]. The mechanism that may explain this teratogen effect in susceptible embryos is, probably, a low concentration of antioxidant enzymes, in face of an increased intrauterine ROS generation due to the hyperglycemic intrauterine environment [34]. Indeed, studies on the post-placentation period have shown that lipoperoxidation is higher in the placenta and in the decidua than in fetuses, suggesting a potential role of placenta to protect fetuses from OS.

While the maternal and fetal complications associated with IUGR, PE and GDM are possible risk factors for induced preterm birth, the leading etiological cause of spontaneous preterm birth (sPTB) is chorioamnionitis, with the ensuing inflammation and OS. Being the pathogenesis completely different, we should distinguish between sPTB versus premature rupture of membranes (pPROM) and preterm labor. Unfortunately, most of the available literature generically links OS with sPTB [35], but only few studies have applied this classification. A specific role of ROS in determining pPROM has been suggested. In particular, increased ROS levels due to maternal infections or inflammation can damage the collagen in the chorioamnion, and the ensuing collagen degradation may contribute to the tearing of the amniotic membranes [36].

Some authors have found that, in spontaneous pPROM cases, pro-inflammatory stimuli such as bacterial lipopolysaccharide (LPS) activate the Toll-like receptors (TLRs), which are responsible for the induction of the nuclear factor kappa-light-chain-enhancer of activated B-cells (NFkB) [37]. This signaling pathway is able to activate the cellular expressions of genes and molecules involved in the inflammatory response, such as the proinflammatory cytokines TNF-alpha, interleukin (IL)-1, IL-12 and chemokines such as interferon-inducible protein 10 and RANTES [38]. This inflammatory environment increases the production, in the fetal brain, of inflammatory cytokines and free radicals which may damage the neural cells in a very critical developmental stage, when fetal antioxidant systems are still immature. Other studies have highlighted that, in sPTB without pPROM, OS-associated prostaglandins such as F2 isoprostane were not elevated compared to sPTB with pPROM, underlying the possible role of inflammation-induced OS in mediating a premature labour onset [39]. Furthermore, a more recent study has shown characteristic patterns of OS-induced DNA damage and senescence in fetal membranes and amniotic fluid in sPTB with pPROM compared with sPTB with intact membranes, further supporting a specific role of OS in pPROM and ensuing preterm labour [40].

Consistently with the Barker hypothesis on the fetal origin of later diseases, by inducing specific DNA modifications, antenatal exposure to OS has also been hypothesized to be involved in several disorders of adult life, including cancer, metabolic disorders and cardiovascular diseases [41]. However, the impact of OS on such long-term outcomes can be modulated by specific factors, such as maternal diet and microbiome. As an example, in experimental models, low-protein and high-fat maternal intakes during pregnancy have been associated with a high susceptibility to OS and with an enhanced oxidative status in the offspring [42,43] and may be potentially associated with such generational sequelae as neurodevelopmental disorders, hypertension, increased infertility rates and diabetes [44,45,46,47,48]. Moreover, an inadequate diet during pregnancy can contribute to an unfavorable microbiome in dams and offspring [49,50,51], which has been further associated with adverse long-term outcomes related to fetal programming [52,53].

## 3. OS and Prematurity-Related Diseases

**Figure 2:**
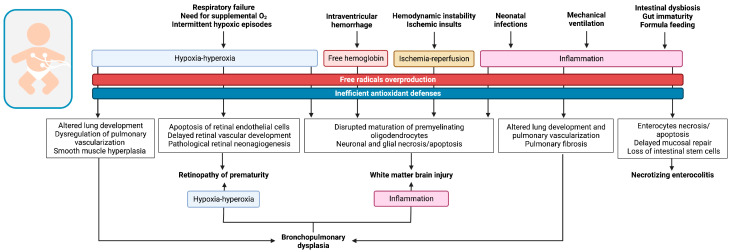
Graphical illustration of the oxidative pathophysiological pathways involved in the development of the main prematurity-related diseases.

As previously discussed, preterm birth is responsible for a sudden translation to the oxygen-rich extrauterine environment while the immature antioxidant defenses of prematurely born neonates are still ineffective in counteracting the oxidative burden that characterizes early postnatal phases, leading to the development of OS. In this contest, OS can be further triggered by specific pathophysiological mechanisms, illustrated in Figure 2, which finally contribute to the pathogenesis of the so-called OS-related diseases of the newborn, addressed in the following paragraphs, by interfering with the physiological development of organs and systems and causing irreversible injuries at a tissue level.

### 3.1. Respiratory Diseases of Preterm Infants

Respiratory distress syndrome (RDS) still represents a significant morbidity for preterm infants. Although clinical management has improved over time, thus reducing the mortality rates directly related to RDS, its chronic consequences, mostly bronchopulmonary dysplasia (BPD), remain unacceptably high [54]. BPD is estimated to affect almost 50% infants below 29 weeks’ GA, and is associated with increased mortality, respiratory morbidity, neurodevelopmental impairment and high healthcare costs [55].

OS plays a significant role in the pathogenesis of lung injury in preterm infants, both in the acute inflammatory insult which characterizes RDS, and in the chronic structural and functional pulmonary alterations typical of BPD. In addition, OS is involved in the pathogenesis of neonatal pulmonary hypertension (PH), which may further worsen the clinical course of BPD [56].

In addition to the inefficiency of preterm infants’ antioxidant defense system and to the high exposure of this population to free radicals, additional factors contribute to increase the susceptibility of the immature preterm lung to OS damage, such as infections, mechanical ventilation and excessive oxygen exposure.

The fetal lung develops in a hypoxic environment: low oxygen tension in utero is essential for the activity of the hypoxia-inducible factor (HIF) family of transcription factors, whose main role is to maintain oxygen homeostasis. Through regulation of over 2500 genes, HIF modulates the expression of hundreds of messenger RNAs, leading to either an increase in oxygen delivery or a decrease in oxygen consumption [57]. HIF-1, which is the most studied factor of the family, plays a key role in fetal oxygen homeostasis through a series of mechanisms balancing oxygen exposure and ROS production. To increase oxygen delivery, HIF-1 regulates erythropoiesis by activating erythropoietin transcription and angiogenesis, serving as a stimulus for angiogenic cytokines and growth factors such as vascular endothelial growth factor (VEGF). In addition, HIF-1 modulates the expression of genes which control the rapid metabolic switch from oxidative to glycolytic metabolism, which is aimed to reduce oxygen consumption, and also improves the efficiency of electron transport, thus reducing mitochondrial oxygen consumption.

Following preterm birth, the exposure of preterm infants to the normoxic or even hyperoxic environment inhibits HIF-1 function: the proteasomal degradation of HIF-1 induced by increased oxygen exposure compromises lung growth and normal alveolarization, and the arrest in cell-cycle results in vascular pruning [58]. Consistently, studies in rodents demonstrate that postnatal oxygen exposure alters the expression of numerous genes which are involved in central pathways of lung development; superimposing intermittent hypoxic episodes (IHE), which are common in preterm infants and constitute a risk factor for BPD [59], further dysregulate the expression of genes involved in lung growth, repair and inflammation and impair antioxidant defense mechanisms [57]. In addition to that, hyperoxia after birth induces epigenetic changes in over 1000 genes involved in lung growth and differentiation, such as TGF-β signaling, whose effects can persist even after the exposure to hyperoxia ceases [60].

Hyperoxia also leads to mitochondrial dysfunction, along with ROS accumulation, induction of a pro-inflammatory response and damage to the alveolar epithelium. Moreover, when superimposed to hypoxia as in the case of IHE, it has been associated with a severe dysfunction of lung endothelial cells, mediated by OS-related mitochondrial damage, which contributes to the development of an abnormal lung vascular phenotype typical of PH [61]. In this respect, mitochondria-targeted antioxidants and inhibition of mitochondrial ROS production were both studied in preclinical models as promising tools for reducing ROS-induced lung damage [57].

ROS production impairs nitric oxide (NO) synthesis, which in turn stimulates an altered growth of the lung smooth muscle and the remodeling of pulmonary vasculature. Strategies aimed at restoring NO function have been found to be effective in improving the growth of lung vessels, and in reducing pulmonary inflammation through the downregulation of proinflammatory genes, adding up a pro-angiogenetic and anti-inflammatory role to the traditional therapeutic action of inhaled NO, which is mainly used for the treatment of acute neonatal PH. The last mechanism through which OS impairs lung growth function is related to inflammation, as ROS stimulate a proinflammatory cascade in the preterm lung, which induces a direct injury to the alveoli, impairs the function of surfactant and disrupts mesenchymal stem cell function [62].

OS is thought to play a central role in acute preterm lung injury as it has been related to several conditions that predispose to RDS, such as maternal diabetes, infection and inflammation and also to management strategies requested for RDS treatment, including oxygen, ventilation and surfactant replacement [63,64]. It has been suggested that an imbalance between OS and antioxidant mechanisms could be a leading mechanisms of injury in the preterm lung: compared to healthy controls, preterm infants with RDS have significantly increased protein oxidation, oxidative DNA damage and lipid peroxidation and also lower markers of oxidative defenses detected on umbilical cord blood [65]. Apparently, the imbalance between OS markers and antioxidant capacity is proportional to the degree of immaturity and is directly related to RDS severity and mortality [66,67]. Interestingly, surfactant replacement, which is a common practice for treating RDS, seems to improve total antioxidant capacity in preterm infants with RDS [66]. Although mechanical ventilation is often required to treat RDS, especially at extremely low GAs, its use has been linked to various forms of lung injury (such as volutrauma, barotrauma and atelectotrauma) collectively known as ventilation-induced lung injury (VILI). It has been shown that even short periods of mechanical ventilation and moderately high oxygen requirements may lead to lung inflammation, as documented by high levels of pro-inflammatory cytokines detected in infants receiving mechanical ventilation compared to controls [68]. By enhancing ROS production, the inflammatory pathway together with the increased oxygen requirements may burst a noxious oxidative cascade, further worsening lung damage.

Lung injury induced by hyperoxia and mechanical ventilation following RDS may impact chronically on the preterm lung health, leading to BPD. According to most recent consensus, the diagnosis of BPD relies on the need of respiratory support at 36 weeks postmenstrual age in very low GA preterm infants; the disease is then graded according to the type of respiratory support required [69] and/or the need for supplemental oxygen [70]. From a pathophysiological point of view, BPD is characterized by airway, alveolar and vascular changes occurring in the immature lung after preterm birth because of sustained inflammation, extracellular matrix remodeling and alteration in growth factor signaling through which the preterm lung responds to early postnatal injury.

Several factors contribute to chronic lung damage, including VILI, infections and prolonged use of high oxygen concentrations [64]. As for hyperoxia exposure, this leads to increased ROS production which, in turn, induces DNA damage, protein oxidation and lipid peroxidation. The following inflammatory response is characterized by the release of cytokines and other molecular mediators which induce mitochondrial dysfunction, cell cycle arrest, release of abnormal growth and angiogenic factors, release of abnormal matrix proteins and, ultimately, cell death. All these factors are implicated in impaired alveolarization, dysregulation of vascular growth, smooth muscle hyperplasia and pulmonary fibrosis, which are all hallmarks of BPD [71].

While to date there are insufficient data to propose the use of potential antioxidant treatments in RDS and BPD, strategies such as non-invasive respiratory support coupled with early and tailored surfactant administration and a careful management of supplemental oxygen have the potential to limit VILI and the ensuing oxidative damage.

### 3.2. Retinopathy of Prematurity

Retinopathy of prematurity (ROP) is a multiphasic disease that affects the development of the immature retinal vessels in preterm neonates, with an incidence and severity that is inversely related to their birth weight (BW) and GA [72]. Human retinal vascularization begins at approximately 16 weeks’ GA, with an evolution from central to periphery that continues up to 36–40 weeks’ GA. ROP pathogenesis is characterized by a biphasic pattern, with an initial interruption of retinal vascular development and a later aberrant vascular growth with fibrovascular proliferation [73]. The severity of ROP vascular abnormalities is classified according five different stages; while stages I–III can spontaneously regress, stages IV–V are associated with worse ophthalmological outcomes, including vitreous hemorrhage and tractional retinal detachment leading to blindness [74].

The primary role of OS in ROP pathogenesis is largely ascertained and, as such, this condition is included among the main OS-related neonatal diseases [1,11,75,76]. Notably, in addition to extremely low GA, which underpins the primary role of the immature antioxidant defenses, other OS-related conditions such as BPD, necrotizing enterocolitis (NEC) and neonatal sepsis, which can further enhance ROS overproduction by different mechanisms (e.g., inflammation and hypoxia-hyperoxia), have been identified among ROP risk factors [72]. Consistently, significant increased levels of oxidative biomarkers have been described in preterm infants with ROP [75].

The main pathophysiological mechanism underlying ROP development is the alternance between hyperoxia and hypoxia that often occurs both in early phases following preterm birth because of RDS, IHE, high oxygen saturation targets or in association with BPD development. In utero, retinal vasculature physiologically develops in an environment characterized by low oxygen tension. Following preterm birth, the exposure to the oxygen-rich extrauterine environment, which is further enhanced by oxygen supplementation, inhibits the growth of retinal vessels by reducing the levels of VEGF and other angiogenic factors. In animal models, this vaso-obliterative process of retinal vasculature seems to be mediated by OS-induced endothelial cells apoptosis [77,78]. The immaturity of other mechanisms involved in vascular regulation, such as those regulated by prostaglandins, PaCO_2_ and NO, can further alter the delicate oxygen balance of the developing retinal vessels, contributing to interrupt the physiological retinal vascularization [79,80]. As the retina matures, the related metabolic demand increases and, as a consequence of the altered vascularization, hypoxia may result. Exogenous factors, mainly related to the infants’ respiratory status (e.g., RDS, BPD, IHE), may further worsen retinal hypoxia. This hypoxic phase promotes the expression of HIFs which, by binding DNA, induces the transcription of angiogenic genes, such as erythropoietin and VEGF, to improve retinal perfusion [80]. HIF transcription can be stabilized by oxidative compounds or inflammatory cytokines, mediated by NFkB pathway, thus further enhancing an aberrant vascular proliferation [81]. The OS ensuing from the alternance of hypoxia and hyperoxia can also lead to a dysfunctional activation of endothelial nitric oxide synthase (eNOS), which generates high amounts of RNS (uncoupling) and, to a lesser extent, of NO. By inducing a compensatory vasodilation to improve blood and oxygen supply to the hypoxic retina, NO contributes to trigger the formation of ROS, which may activate angiogenic signaling pathways through VEGF or nicotinamide-adenine-dinucleotide phosphate oxidase [82]. Furthermore, experimental evidence has shown that isoprostanes, which are derived by OS-mediated oxidation of arachidonic acid, may contribute to enhance the endothelial damage by inducing thromboxane A2 production [83,84].

IHE perfectly reproduce the pathophysiological model of hypoxia/hyperoxia underlying ROP development. Data from both animal and clinical studies have demonstrated how specific oxygen fluctuation patterns play a major role in ROP severity. In murine models, clustered IHE were associated with increased levels of systemic and ocular VEGF and resulted in a more severe form of retinopathy when compared to dispersed episodes [85]. Moreover, repeated oxygen fluctuations resulted in a greater expression of a pathologic isoform of VEGF associated with pathologic intravitreous neovascularization [86]. In line with these findings, Di Fiore et al. reported a higher incidence of IHE characterized by increased duration, higher nadir and increased time variability between the events in preterm infants with more severe ROP stages [87,88]. Furthermore, in a rat model of retinopathy, a protective effect of caffeine, which exerts a preventive effect towards hypoxic spells, towards a severe disease has been reported [89].

### 3.3. Prematurity-Related Brain Injury

The noxious role of OS in the development of neurological complications of prematurity is well-established [10,90]. The pathogenesis of oxidative brain injury in preterm infants relies in the interaction between the intrinsic vulnerability of the neonatal brain, the inadequate maturation of antioxidant defenses following preterm birth and specific etiopathogenic pathways that ultimately converge on ROS and RNS overproduction. Maturation-dependent factors, such as GA, are important modulators of the consequences of OS on the developing brain, and can influence both the type and the extent of OS-related brain injury. In particular, at lower GAs, not only the antioxidant enzyme systems are highly inefficient to counteract the burden of OS, but the white matter precursors premyelinating oligodendrocytes (pre-OL) are particularly vulnerable to free radicals, which can disrupt their differentiation into mature oligodendrocytes, thus affecting white matter development [91]. In this regard, experimental evidence has shown a reduced expression of differentiation-promoting genes, increased expression of differentiation-inhibiting genes and a persistent histone acetylation in pre-OL exposed to free radicals, thus shedding light on the molecular mechanisms through which OS hinders their maturation [92].

As a consequence of this maturational vulnerability of pre-OL, white matter damage is the type of injury most typically observed as a consequence of OS in the preterm population. Based on its macroscopic and microscopic characteristics, it can be classified as cystic periventricular leukomalacia (PVL), characterized by focal necrotic and apoptotic phenomena in the periventricular white matter that tend to evolve to the formation of confluent macroscopic cysts, or non-cystic PVL, characterized by pre-OL loss and marked astrogliosis and microgliosis, which tend to evolve in microscopic glial scars [93]. Interestingly, the serum concentration of isoprostanes, which are derived from lipid peroxidation, in preterm neonates aged 24–48 h has been recently shown to significantly correlate with the extent of white matter injury at magnetic resonance imaging performed at term-equivalent age [94].

Among the pathophysiological mechanisms underlying the development of oxidative brain injury in preterm neonates, hypoxia-ischemia-reperfusion plays a major role [95]. Cerebral hypoxia-ischemia is characterized by an acute or subacute interruption of blood flow and oxygen delivery to the brain; this affects the oxidative phosphorylation, shifting the cell metabolism to anaerobic. The use of glucose for anaerobic glycolysis, however, is highly inefficient and contributes to the depletion of cerebral glucose, which is the main energy source for neural cells [96]. Hence, the production of adenosine triphosphate (ATP) subsequently decreases and the ATP-dependent ion pumps on cell membranes become inactive, leading to necrotic cell death. By inducing the release of glutamate, the cell membrane depolarization occurring in this phase triggers the so-called glutamate excitotoxicity, which enhances free radical production and activates apoptotic pathways [97]. Following a hypoxic-ischemic insult, reperfusion is fundamental to restore blood and oxygen delivery to the involved tissue. Nevertheless, the increased O_2_ availability ensuing from blood flow restoration not only fuels an overproduction of free radicals in the damaged mitochondrial ETC, but also triggers the up-regulation of pro-oxidant enzymes such as xanthine oxidase, whose proteolytic conversion from xanthine dehydrogenase in energy-depleted cells uses the newly available O_2_ as a cofactor [98]. The oxidative burst triggered by reperfusion further disrupts mitochondrial phosphorylation, feeding a vicious circle that finally results in secondary energy failure and programmed neuronal death [99]. When hypoxia-ischemia-reperfusion occurs in preterm infants, due to their inefficient antioxidant defenses and to the anatomical immaturity of their central nervous system, which includes an increased permeability of the blood–brain barrier (BBB), it further amplifies the noxious effects of OS compared to term peers [100]. Moreover, in the preterm population, the areas which are most typically affected by hypoxia-ischemia-reperfusion are white matter watershed regions, due to their peculiar anatomical features, characterized by the presence of arterial border-zones and end-zones that lay between the penetrating branches of anterior, middle and posterior cerebral arteries, which make them particularly sensible to blood flow fluctuations [93].

Intermittent hypoxia-hyperoxia is an additional important source of OS in the preterm population, with possible neurological implications [101]. Due to their immature respiratory control and to the frequent coexistence of respiratory comorbidities, such as RDS, that require O_2_ supplementation to reach oxygenation targets, preterm infants are particularly prone to IHE. Differently from hypoxia-ischemia-reperfusion, the decreased O_2_ availability at the brain level in this case is mainly driven by hypoxemia rather than ischemia and results in a less profound hypoxic burden. Moreover, the restoration of an adequate O_2_ delivery is often achieved by increasing the fraction of inspired O_2_, which can lead to a transient hyperoxic phase that fuels up ROS and RNS generation. The extent of the ensuing oxidative burst increases with the frequency of hypoxic spells, with repetitive cycles being associated with the development of poorer neural outcomes [102]. Consistently, the use of caffeine for apneas prevention has been associated with enhanced myelination in animals [103] and with an improved micro-structure of white matter in treated preterm infants [104].

Free hemoglobin resulting from hemorrhagic events also represents an important source of free radicals, due to the pro-oxidant capacities of the iron-containing heme prosthetic group which act as a Fenton reagent [105]. A fitting example for this pathogenic mechanism in development of oxidative brain injury is provided by intraventricular hemorrhage (IVH), a common neurological complication of very preterm infants. IVH typically originates in the frail vasculature of germinal matrix; as the bleeding enlarges, the underlying ependyma breaks and the ventricle is filled with blood. The degradation of blood components that follow the primary bleeding releases large amounts of free hemoglobin and other neurotoxic substances that can easily cross the immature BBB, inducing mitochondrial dysfunction [106] and triggering not only inflammatory reactions and glutamate excitotoxicity, but also free radical production in the surrounding regions [107]. The ensuing OS, in turn, causes neuronal and glial apoptosis [108] and disrupts the maturation of pre-OL [109], consistently with the development of periventricular white matter injury frequently observed following severe IVH [110] and with animal evidence showing increased ROS and RNS levels in the periventricular white matter adjacent to the hemorrhaged ventricles [111,112].

Finally, but not less importantly, inflammation plays a key role in oxidative brain injury. The activation of neutrophils, macrophages and lymphocytes in response to infectious or inflammatory stimuli releases large amounts of free radicals and of proteases and cytokines that can further contribute to OS generation [113]. Cystic and non-cystic white matter injury have been largely observed following such inflammatory conditions as maternal chorioamnionitis [114,115], neonatal infections [116] and BPD [117]. The link between inflammation, OS and ensuing brain injury is further supported by autoptic evidence in PVL specimens of higher concentrations of tumor necrosis factor (TNF)-α, interferon-γ, IL-6 and IL-2, increased protein nitration and lipid peroxidation in pre-OL and significant activation of white matter microglia [118,119,120,121].

### 3.4. Necrotizing Enterocolitis

NEC is the most devastating gastrointestinal neonatal disease [122]. NEC occurs at a frequency of 1–3 per 1000 live birth, and almost 90% of the cases affect infants born preterm, the risk being inversely related to BW and GA. Despite advances in perinatal and neonatal care, NEC remains a leading cause of morbidity and mortality in preterm infants, with mortality rates reaching approximately 30% [123].

The pathogenesis of NEC remains poorly understood; available evidence supports a multifactorial mechanism. Prematurity itself is recognized as the main risk factor; the concurrent presence of an immature intestinal tract and dysregulated immune response, abnormal microbial intestinal colonization (intestinal dysbiosis), together with hypoxic-ischemic injury and imbalanced microvascular tone contribute to intestinal inflammation and necrosis [124].

Several studies have suggested a role of OS in the pathogenesis of NEC; Aydemir et al. [4] compared, in preterm infants with and without NEC, the global oxidant/antioxidant status by measuring total antioxidant capacity, total oxidant status (TOS) and oxidative stress index (OSI) [125]. Infants with NEC had significantly higher TOS and OSI levels compared with controls, and increased levels of TOS and OSI were associated with the severity of NEC. Furthermore, Perrone et al. demonstrated a strong association between the concentration of OS markers in cord blood and the occurrence of NEC in preterm infants [126]. According to epidemiologic observations and studies performed in animal models, the pathogenesis of NEC seems to be largely related to the immaturity of the gastrointestinal tract. The intestinal mucosa of preterm infants is exposed to constant injury, triggered by hypoxia, hypothermia, formula feeding etc.

Innate immune receptor TLR4, whose expression is increased in the intestinal tract of preterm newborns, seems to have a central role in NEC pathogenesis [124]. Excessive signaling in the epithelial TLR4 pathway in response to LPS presented by Gram-negative bacteria leads to the loss of enterocytes through apoptosis, followed by delayed repair through inhibition of migration and TLR4-mediated loss of intestinal stem cells. These factors favour the translocation of bacteria and LPS into the circulation and, consequently, the production of proinflammatory cytokines and ROS, increased expression of inducible nitric oxide synthase (iNOS) and impaired perfusion via eNOS dysregulation mediated by TLR4 [127]. Experimental models have provided clues of a direct link between ROS production in the premature gut and NEC. NO seems to play a crucial and ambiguous role in OS-related NEC damage. NO is produced from arginine in a reaction catalyzed in the intestine mainly by two NO synthases, eNOS and iNOS. eNOS is constitutively expressed in the intestinal capillaries and is regulated by NO concentrations, whereas iNOS is mainly located in immune cells and activated by proinflammatory cytokines during inflammation and pathogen response. Sustained upregulation of iNOS in the intestinal mucosa is known to occur in preterm infants during the development of NEC [128]. In a neonatal rat model of NEC, increased concentration of iNOS caused by LPS was found in the intestinal mesentery in the late stage of the disease [129]. This upregulation may contribute to intestinal injury via high levels of NO, which contributes to the formation of peroxynitrite, a RNS highly toxic to epithelial cells [130]. During OS, eNOS switches from producing NO to •O_2_^−^; this switch in enzyme function is called “eNOS uncoupling” and contributes to form additional ROS, exaggerating the uncoupling [131]. Studies on rat models indicate that eNOS uncoupling becomes worse during NEC progression [129].

It is well known that the characteristics of enteral feeding have a strong impact on the risk of NEC in preterm neonates; specifically, it is generally recognized that the risk of NEC is reduced when preterm infants are fed an exclusively human milk (HM)-based diet compared to diets containing bovine-derived products (formula and/or traditional HM fortifiers) [132]; the detrimental effect of bovine-based products appears to be related to an increased intestinal permeability, a direct toxic effect on the gut epithelial cells and to an upregulation of OS [133]. Friel et al. demonstrated that HM, provided by mothers of both term and preterm infants, has better antioxidant properties than formula and that preterm and term HM have equal resistance to OS [134]. The same authors also examined the effect of HM fortification on OS markers in preterm infants and found that those fed with HM plus HM fortifier had the highest urinary levels of F2-isoprostanes, compared to both infants fed with exclusive HM and formula [135]. This finding, which has not been further explored, might explain, at least partially, the beneficial effect of HM diets without any bovine-derived supplement on NEC incidence in preterm infants. Among HM components, human milk oligosaccharides (HMO) have shown to modulate NO pathway. Good et al. evaluated the role of 2′-fucosyllactose (HMO-2′ FL), an abundant HMO, in a NEC rat model [136]. Their data showed that the addition of HMO-2′ FL to milk formula reduced the severity of experimental NEC; specifically, HMO-2′ FL protective effects occurred via restoration of intestinal perfusion through upregulation of eNOS and downregulation of proinflammatory molecules including iNOS [136]. It has also been proposed that the underlying initial condition in NEC pathogenesis is the reduced ability of the neonatal gut epithelial cells (NGECs) to clear OS when exposed to enteral feeding [137]. An agent-based computational model has demonstrated that impaired OS management can lead to apoptosis and inflammation of NGECs when additional bacterial TLR4 activation occurs.

The role of intestinal dysbiosis is emerging as a major pathogenetic factor for NEC [138]. NEC does not have a specific microbial signature; however, large-scale variation in bacterial taxa at high phylogenetic level was reported to precede NEC [139,140]. The excessive signaling in the TLR4 pathway in response to LPS presented by Gram-negative bacteria, modulating host–microbiota interaction, has often been implicated in NEC onset. Recent data also suggest that variation in gut microbiota diversity and composition induced by different feeding practices could be associated with the extent of systemic OS, measured as levels of urinary F2-isoprostane metabolites in very low birth weight preterm infants [141]. Moreover, it has been demonstrated that fecal microbiota transplantation is effective in a mouse model of NEC through OS modulation and reduced TLR4-mediated colonic inflammation [142,143]. Fecal microbiota transplantation eliminated superoxide production and promoted NO production, contrasting eNOS uncoupling.

Taken together, available data suggest that OS may act as a downstream amplifying component in the inflammatory cascade, which results in intestinal injury and may trigger NEC development.

### 3.5. Neonatal Infections

A delicate balance between beneficial and toxic effects of free radicals exists in infectious diseases. ROS and RNS play an important role in the host defense system during infections, with several cells of the innate immune system—neutrophils, macrophages and monocytes—releasing these compounds to destroy invading pathogens. A typical example of the importance of this mechanism is represented by the impaired production of free radicals as seen in granulomatous disease, which manifests with persistent and multiple infections.

However, the activation of the immune system during a systemic infection is also paralleled by a complex chain of redox events, maintained by the production of a significant amount of ROS and RNS, which may generate OS with a deleterious process involving most cell structures, mediated by the activation of DNA-transcription processes, and leading to mitochondrial functional impairment.

Most neonatal studies on the relationship between infections and OS have been focused on neonatal sepsis. Neonatal sepsis is a clinical syndrome characterized by both infection and systemic inflammatory response syndrome [144], with a high morbidity and mortality in the preterm population [145]. Infection acts as the trigger of a complex host response which includes both inflammatory and oxidative mechanisms. The inflammatory cascade is mainly initiated by IL-6 and IL-8, which have been demonstrated to be increased in the first 24 h after sepsis onset in both early onset sepsis (EOS) and late onset sepsis (LOS) [146,147]. However, a differential cytokine expression profile and different pathways could be involved in the initiation and maintenance of the sepsis process according to the timing of sepsis presentation and to the neonate’s GA, with several pro-inflammatory molecules (e.g., TNF-α, interferon-γ) being up-regulated only in the most mature neonates [148,149]. The release of pro-inflammatory cytokines triggers the initiation of OS-related pathways, in which NF-kB transcription and the direct cytokine-induced activation of the NADPH oxidase constitute key factors [146]. In particular, NF-kB activates the transcription of several genes with the final effect of an increased production of NO and superoxide, whereas the direct activation of the NADPH further increases superoxide levels. These observations have been confirmed both in experimental models and in human studies, the latter demonstrating an increased level of NO in septic neonates [150,151,152]. Once again, NO production in the setting of bacteremia seems to be different according to GA, with the most immature babies (<27 weeks’ GA) having lower basal NO levels, but higher NO levels during the first 48 h of bacteremia as compared with more mature infants [151]. The final effect of the activation of this sepsis-related redox pathway on the host is mitochondrial dysfunction due to a direct ETC inhibition by ROS and RNS [146,149]. Indeed, even though also antioxidant defenses are induced, during neonatal sepsis there is an unbalance favoring OS.

As already mentioned, the detrimental effect of sepsis on the neonatal redox status is more evident in the setting of prematurity, due to the pro-oxidant characteristics of preterm infants (e.g., impaired antioxidant capacity, antenatal and postnatal exposure to several factors associated with OS). It has been proposed that this redox imbalance characterized by a prevalence of pro-oxidant pathways over antioxidants defenses might be implicated not only in the immediate cellular effects but also in some of the long-term morbidity related to sepsis, such as long-term neurodevelopment sequelae [149].

The clinical application of this knowledges has led to conflicting results. Several markers of OS and of antioxidant defenses have been studied in term and preterm neonates, both in the attempt of predicting EOS using cord blood samples [153,154,155,156], diagnosing sepsis [157,158] and assessing severity in both EOS and LOS [159,160,161]. Even though the major role of OS in the pathogenesis of sepsis has been clearly demonstrated, results on single biomarkers might not be considered conclusive. Moreover, to date, no relationship has been established between OS markers and long-term neurodevelopmental outcomes in both term and preterm septic neonates. Similarly, the usefulness of antioxidants as adjunctive treatments in neonatal sepsis needs to be further assessed in rigorous well-designed randomized studies.

OS pathways seem also to play a role in the host response to viral respiratory infections. Experimental data demonstrate that respiratory syncytial virus both actively induces OS and down regulates components of the antioxidant pathways, and that OS might itself have a pro-viral effect [162]. Similar data have been reported also following influenza infection [163]. Even though no data are currently available for preterm neonates, it is known that neonates and particularly those born preterm exhibit an increased susceptibility to respiratory viral infections. The possible development of specific treatments to limit the high burden of disease related to these infections in preterm neonates represent a hot topic for future research in neonatology.

## 4. Conclusions

The exposure to the detrimental effects of OS can start as soon as after conception and plays a major role in the development of common pregnancy disorders, such as IUGR, GDM or PE, which, by triggering hypoxic-ischemic or inflammatory cascades, can further worsen the intrauterine oxidative burden. Maternal chorioamnionitis, which is a leading cause of sPTB, has also been associated with an enhanced oxidative status, driven by the underlying inflammatory responses. Hence, both the development and the course of these antenatal conditions, including their progression and the related pregnancy and neonatal outcomes, can be worsened by OS.

On the fetal side, antioxidant systems are still immature and the effects of OS exposure during antenatal life can therefore have long-term negative effects on neonatal outcomes. When preterm birth occurs, the inefficient antioxidant defenses together with the exposure to several pro-oxidative triggers such as inflammation, hyperoxia, hypoxia-ischemia, altered vascular regulation and the anatomical and functional immaturity of organs and systems, make preterm infants particularly susceptible to multisystemic oxidative damage.

Exposure to OS during fetal and early postnatal phases may contribute to disrupt the physiological lung development and to enhance the pathogenic cascades that finally lead to BPD. The consequences of OS on neonatal lung disease are largely established especially for chronic conditions, such as BPD, which is the result of the chronic evolution of neonatal acute RDS worsened by postnatal lung injury related to several conditions and treatments in which OS might play a role (i.e., sepsis and VILI). ROP provides a typical example of the noxious pathogenic mechanisms related to OS; in particular, several experimental and clinical studies have confirmed the association between ROP and OS not only for the disease onset, but also for its severity. Specific pathophysiological pathways resulting from the combination of OS and the immaturity of the developing brain and gut have been identified in the development of white matter brain damage or NEC, typical complications of prematurity. With regard to the latter condition, experimental data have also suggested a possible worsening role of OS on the course of the disease. Eventually, the oxidative burst elicited by immune responses in response to infective complications may further disrupt the fragile redox balance of preterm neonates, and has been associated with the severity of OS-driven injury at a multiorgan level. Awareness of the antenatal and postnatal mechanisms responsible for OS in preterm infants may increase awareness on potential strategies aimed at preventing the so-called OS-related diseases in this fragile population, or at reducing the ensuing clinical burden.

## Data Availability

Not applicable.

## References

[1] Ozsurekci Y., Aykac K. (2016). Oxidative Stress Related Diseases in Newborns. Oxidative Med. Cell. Longev..

[2] Lushchak V.I. (2014). Free radicals, reactive oxygen species, oxidative stress and its classification. Chem. Biol. Interact..

[3] Cobb C.A., Cole M.P. (2015). Oxidative and nitrative stress in neurodegeneration. Neurobiol. Dis..

[4] Lushchak V.I. (2012). Glutathione Homeostasis and Functions: Potential Targets for Medical Interventions. J. Amino Acids.

[5] Marseglia L., D’Angelo G., Manti S., Arrigo T., Barberi I., Reiter R.J., Gitto E. (2014). Oxidative Stress-Mediated Aging during the Fetal and Perinatal Periods. Oxidative Med. Cell. Longev..

[6] Davis J.M., Auten R.L. (2010). Maturation of the antioxidant system and the effects on preterm birth. Semin. Fetal Neonatal Med..

[7] Folkerth R.D., Haynes R.L., Borenstein N.S., Belliveau R.A., Trachtenberg F., Rosenberg P.A., Volpe J.J., Kinney H.C. (2004). Developmental Lag in Superoxide Dismutases Relative to Other Antioxidant Enzymes in Premyelinated Human Telencephalic White Matter. J. Neuropathol. Exp. Neurol..

[8] Thibeault D.W. (2000). The precarious antioxidant defenses of the preterm infant. Am. J. Perinatol..

[9] Ferrante G., Montante C., Notarbartolo V., Giuffrè M. (2022). Antioxidants: Role the in prevention and treatment of bronchopulmonary dysplasia. Paediatr. Respir. Rev..

[10] Martini S., Castellini L., Parladori R., Paoletti V., Aceti A., Corvaglia L. (2021). Free Radicals and Neonatal Brain Injury: From Underlying Pathophysiology to Antioxidant Treatment Perspectives. Antioxidants.

[11] Lembo C., Buonocore G., Perrone S. (2021). Oxidative Stress in Preterm Newborns. Antioxidants.

[12] Falsaperla R., Lombardo F., Filosco F., Romano C., Saporito M.A.N., Puglisi F., Piro E., Ruggieri M., Pavone P. (2020). Oxidative Stress in Preterm Infants: Overview of Current Evidence and Future Prospects. Pharmaceuticals.

[13] Sultana Z., Maiti K., Aitken R.J., Morris J., Dedman L., Smith R. (2017). Oxidative stress, placental ageing-related pathologies and adverse pregnancy outcomes. Am. J. Reprod. Immunol..

[14] Pijnenborg R., Dixon G., Robertson W., Brosens I. (1980). Trophoblastic invasion of human decidua from 8 to 18 weeks of pregnancy. Placenta.

[15] Watson A.L., Skepper J.N., Jauniaux E., Burton G.J. (1998). Susceptibility of Human Placental Syncytiotrophoblastic Mitochondria to Oxygen-Mediated Damage in Relation to Gestational Age. J. Clin. Endocrinol. Metab..

[16] Genbacev O., Zhou Y., Ludlow J.W., Fisher S.J. (1997). Regulation of Human Placental Development by Oxygen Tension. Science.

[17] Jauniaux E., Watson A.L., Hempstock J., Bao Y.-P., Skepper J.N., Burton G.J. (2000). Onset of Maternal Arterial Blood Flow and Placental Oxidative Stress: A Possible Factor in Human Early Pregnancy Failure. Am. J. Pathol..

[18] Jauniaux E., Hempstock J., Greenwold N., Burton G.J. (2003). Trophoblastic Oxidative Stress in Relation to Temporal and Regional Differences in Maternal Placental Blood Flow in Normal and Abnormal Early Pregnancies. Am. J. Pathol..

[19] Tsonis O., Balogun S., Adjei J.O., Mogekwu O., Iliodromiti S. (2021). Management of recurrent miscarriages: An overview of current evidence. Curr. Opin. Obstet. Gynecol..

[20] Simşek M., Naziroğlu M., Simşek H., Cay M., Aksakal M., Kumru S. (1998). Blood plasma levels of lipoperoxides, glutathione peroxidase, beta carotene, vitamin A and E in women with habitual abortion. Cell Biochem. Funct..

[21] Biri A., Bozkurt N., Turp A., Kavutcu M., Himmetoglu Ö., Durak I. (2007). Role of Oxidative Stress in Intrauterine Growth Restriction. Gynecol. Obstet. Investig..

[22] American College of Obstetricians and Gynecologists (2019). ACOG Practice Bulletin No. 204: Fetal Growth Restriction. Obstet. Gynecol..

[23] Chappell L.C., Cluver C.A., Kingdom J., Tong S. (2021). Pre-eclampsia. Lancet.

[24] Siddiqui I.A., Jaleel A., Tamimi W., Al Kadri H.M.F. (2010). Role of oxidative stress in the pathogenesis of preeclampsia. Arch. Gynecol. Obstet..

[25] Sankaralingam S., Arenas I.A., Lalu M.M., Davidge S.T. (2006). Preeclampsia: Current understanding of the molecular basis of vascular dysfunction. Expert Rev. Mol. Med..

[26] American College of Obstetricians and Gynecologists (2018). ACOG Practice Bulletin No. 190: Gestational Diabetes Mellitus. Obstet. Gynecol..

[27] Phoswa W.N., Khaliq O.P. (2021). The Role of Oxidative Stress in Hypertensive Disorders of Pregnancy (Preeclampsia, Gestational Hypertension) and Metabolic Disorder of Pregnancy (Gestational Diabetes Mellitus). Oxidative Med. Cell. Longev..

[28] Arribas L., Almansa I., Miranda M., Muriach M., Romero F.J., Villar V.M. (2016). Serum Malondialdehyde Concentration and Glutathione Peroxidase Activity in a Longitudinal Study of Gestational Diabetes. PLoS ONE.

[29] Biri A., Onan A., Devrim E., Babacan F., Kavutcu M., Durak I. (2006). Oxidant Status in Maternal and Cord Plasma and Placental Tissue in Gestational Diabetes. Placenta.

[30] Coughlan M., Vervaart P., Permezel M., Georgiou H., Rice G. (2004). Altered Placental Oxidative Stress Status in Gestational Diabetes Mellitus. Placenta.

[31] Rudge M.V.C., Costa E., Barbisan L.F., Damasceno D.C., Bueno A., Saito F.H., Calderon I., Rodrigues M.M.P. (2012). Evaluation of cell proliferation and apoptosis in placentas of rats with severe diabetes. Braz. Arch. Biol. Technol..

[32] Correa A., Gilboa S.M., Besser L.M., Botto L.D., Moore C.A., Hobbs C.A., Cleves M.A., Riehle-Colarusso T.J., Waller D.K., Reece E.A. (2008). Diabetes mellitus and birth defects. Am. J. Obstet. Gynecol..

[33] Tinker S.C., Gilboa S.M., Moore C.A., Waller D.K., Simeone R.M., Kim S.Y., Jamieson D.J., Botto L.D., Reefhuis J. (2020). Specific birth defects in pregnancies of women with diabetes: National Birth Defects Prevention Study, 1997–2011. Am. J. Obstet. Gynecol..

[34] Weksler-Zangen S., Yaffe P., Ornoy A. (2003). Reduced SOD activity and increased neural tube defects in embryos of the sensitive but not of the resistant Cohen diabetic rats cultured under diabetic conditions. Birth Defects Res..

[35] Moore T.A., Ahmad I.M., Zimmerman M.C. (2018). Oxidative Stress and Preterm Birth: An Integrative Review. Biol. Res. Nurs..

[36] Woods J. (2001). Reactive Oxygen Species and Preterm Premature Rupture of Membranes—A Review. Placenta.

[37] Kumar N., Nandula P., Menden H., Jarzembowski J., Sampath V. (2017). Placental TLR/NLR expression signatures are altered with gestational age and inflammation. J. Matern. Fetal Neonatal Med..

[38] Hoffmann A., Baltimore D. (2006). Circuitry of nuclear factor kappaB signaling. Immunol. Rev..

[39] Menon R., Fortunato S.J., Milne G.L., Brou L.M., Carnevale C.M., Sanchez S.C.M., Hubbard L.B., Lappas M., Drobek C.O.B., Taylor R.N. (2011). Amniotic Fluid Eicosanoids in Preterm and Term Births: Effects of Risk Factors for Spontaneous Preterm Labor. Obstet. Gynecol..

[40] Dutta E.H., Behnia F., Boldogh I., Saade G.R., Taylor B.D., Kacerovský M., Menon R. (2016). Oxidative stress damage-associated molecular signaling pathways differentiate spontaneous preterm birth and preterm premature rupture of the membranes. Mol. Hum. Reprod..

[41] Indrio F., Martini S., Francavilla R., Corvaglia L., Cristofori F., Mastrolia S.A., Neu J., Rautava S., Spena G.R., Raimondi F. (2017). Epigenetic Matters: The Link between Early Nutrition, Microbiome, and Long-term Health Development. Front. Pediatr..

[42] Moraes-Souza R.Q., Vesentini G., Paula V.G., Sinzato Y.K., Soares T.S., Gelaleti R.B., Volpato G.T., Damasceno D.C. (2021). Oxidative Stress Profile of Mothers and Their Offspring after Maternal Consumption of High-Fat Diet in Rodents: A Systematic Review and Meta-Analysis. Oxidative Med. Cell. Longev..

[43] de Sousa S.M., Braz G.R.F., Freitas C.D.M., de Santana D.F., Sellitti D.F., Fernandes M.P., Lagranha C.J. (2018). Oxidative injuries induced by maternal low-protein diet in female brainstem. Nutr. Neurosci..

[44] Tain Y.-L., Hsu C.-N. (2022). Maternal High-Fat Diet and Offspring Hypertension. Int. J. Mol. Sci..

[45] Edlow A.G. (2017). Maternal obesity and neurodevelopmental and psychiatric disorders in offspring. Prenat. Diagn..

[46] Sinzato Y.K., Paula V.G., Gallego F.Q., Moraes-Souza R.Q., Corrente J.E., Volpato G.T., Damasceno D.C. (2022). Maternal Diabetes and Postnatal High-Fat Diet on Pregnant Offspring. Front. Cell Dev. Biol..

[47] Pedroza A., Ferreira D.S., Santana D.F., da Silva P.T., de Aguiar Júnior F.C.A., Sellitti D.F., Lagranha C.J. (2019). A maternal low-protein diet and neonatal overnutrition result in similar changes to glomerular morphology and renal cortical oxidative stress measures in male Wistar rats. Appl. Physiol. Nutr. Metab..

[48] Tarry-Adkins J.L., Chen J., Jones R.H., Smith N.H., Ozanne S.E. (2010). Poor maternal nutrition leads to alterations in oxidative stress, antioxidant defense capacity, and markers of fibrosis in rat islets: Potential underlying mechanisms for development of the diabetic phenotype in later life. FASEB J..

[49] Sikalidis A.K., Maykish A. (2020). The Gut Microbiome and Type 2 Diabetes Mellitus: Discussing A Complex Relationship. Biomedicines.

[50] Hu C., Yan Y., Ji F., Zhou H. (2021). Maternal Obesity Increases Oxidative Stress in Placenta and It Is Associated with Intestinal Microbiota. Front. Cell. Infect. Microbiol..

[51] Mazenc A., Mervant L., Maslo C., Lencina C., Bézirard V., Levêque M., Ahn I., Alquier-Bacquié V., Naud N., Héliès-Toussaint C. (2022). Maternal heme-enriched diet promotes a gut pro-oxidative status associated with microbiota alteration, gut leakiness and glucose intolerance in mice offspring. Redox Biol..

[52] Wang Y.W., Yu H.R., Tiao M.M., Tain Y.L., Lin I.C., Sheen J.M., Lin Y.J., Chang K.A., Chen C.C., Tsai C.C. (2021). Maternal Obesity Related to High Fat Diet Induces Placenta Remodeling and Gut Microbiome Shaping That Are Responsible for Fetal Liver Lipid Dysmetabolism. Front. Nutr..

[53] Gao Y., Nanan R., Macia L., Tan J., Sominsky L., Quinn T.P., O’Hely M., Ponsonby A.-L., Tang M.L., Collier F. (2021). The maternal gut microbiome during pregnancy and offspring allergy and asthma. J. Allergy Clin. Immunol..

[54] Sweet D.G., Carnielli V., Greisen G., Hallman M., Ozek E., Pas A.T., Plavka R., Roehr C.C., Saugstad O.D., Simeoni U. (2019). European Consensus Guidelines on the Management of Respiratory Distress Syndrome–2019 Update. Neonatology.

[55] Gilfillan M., Bhandari A., Bhandari V. (2021). Diagnosis and management of bronchopulmonary dysplasia. BMJ.

[56] Cannavò L., Perrone S., Viola V., Marseglia L., Di Rosa G., Gitto E. (2021). Oxidative Stress and Respiratory Diseases in Preterm Newborns. Int. J. Mol. Sci..

[57] Choi Y., Rekers L., Dong Y., Holzfurtner L., Goetz M.J., Shahzad T., Zimmer K.-P., Behnke J., Behnke J., Bellusci S. (2021). Oxygen Toxicity to the Immature Lung—Part I: Pathomechanistic Understanding and Preclinical Perspectives. Int. J. Mol. Sci..

[58] Saugstad O.D., Oei J.-L., Lakshminrusimha S., Vento M. (2019). Oxygen therapy of the newborn from molecular understanding to clinical practice. Pediatr. Res..

[59] Jensen E.A., Whyte R.K., Schmidt B., Bassler D., Vain N.E., Roberts R.S., Shah P., Brown L., Wenger L., Frye S. (2021). Association between Intermittent Hypoxemia and Severe Bronchopulmonary Dysplasia in Preterm Infants. Am. J. Respir. Crit. Care Med..

[60] Bik-Multanowski M., Revhaug C., Grabowska A., Dobosz A., Madetko-Talowska A., Zasada M., Saugstad O.D. (2018). Hyperoxia induces epigenetic changes in newborn mice lungs. Free. Radic. Biol. Med..

[61] Damianos A., Kulandavelu S., Chen P., Nwajei P., Batlahally S., Sharma M., Alvarez-Cubela S., Domínguez-Bendala J., Zambrano R., Huang J. (2022). Neonatal intermittent hypoxia persistently impairs lung vascular development and induces longterm lung mitochondrial DNA damage. J. Appl. Physiol..

[62] Gronbach J., Shahzad T., Radajewski S., Chao C.-M., Bellusci S., Morty R.E., Reicherzer T., Ehrhardt H. (2018). The Potentials and Caveats of Mesenchymal Stromal Cell-Based Therapies in the Preterm Infant. Stem Cells Int..

[63] Ferrante G., Carota G., Volti G.L., Giuffrè M. (2021). Biomarkers of Oxidative Stress for Neonatal Lung Disease. Front. Pediatr..

[64] Marseglia L., D’Angelo G., Granese R., Falsaperla R., Reiter R.J., Corsello G., Gitto E. (2019). Role of oxidative stress in neonatal respiratory distress syndrome. Free. Radic. Biol. Med..

[65] Negi R., Pande D., Karki K., Kumar A., Khanna R.S., Khanna H.D. (2015). A novel approach to study oxidative stress in neonatal respiratory distress syndrome. BBA Clin..

[66] Dizdar E.A., Uras N., Oguz S., Erdeve O., Sari F.N., Aydemir C., Dilmen U. (2011). Total antioxidant capacity and total oxidant status after surfactant treatment in preterm infants with respiratory distress syndrome. Ann. Clin. Biochem..

[67] Elkabany Z.A., El-Farrash R.A., Shinkar D.M., Ismail E.A., Nada A.S., Farag A.S., Elsayed M.A., Salama D.H., Macken E.L., Gaballah S.A. (2020). Oxidative stress markers in neonatal respiratory distress syndrome: Advanced oxidation protein products and 8-hydroxy-2-deoxyguanosine in relation to disease severity. Pediatr. Res..

[68] Carvalho C.G., Procianoy R.S., Neto E.C., Silveira R.C. (2018). Preterm Neonates with Respiratory Distress Syndrome: Ventilator-Induced Lung Injury and Oxidative Stress. J. Immunol. Res..

[69] Jensen E.A., Dysart K., Gantz M.G., McDonald S., Bamat N.A., Keszler M., Kirpalani H., Laughon M.M., Poindexter B.B., Duncan A.F. (2019). The Diagnosis of Bronchopulmonary Dysplasia in Very Preterm Infants. An Evidence-based Approach. Am. J. Respir. Crit. Care Med..

[70] Higgins R.D., Jobe A.H., Koso-Thomas M., Bancalari E., Viscardi R.M., Ha rtert T.V., Ryan R.M., Kallapur S.G., Steinhorn R.H., Konduri G.G. (2018). Bronchopulmonary Dysplasia: Executive Summary of a Workshop. J. Pediatr..

[71] Sahni M., Bhandari V. (2021). Patho-mechanisms of the origins of bronchopulmonary dysplasia. Mol. Cell. Pediatr..

[72] Yucel O.E., Eraydin B., Niyaz L., Terzi O. (2022). Incidence and risk factors for retinopathy of prematurity in premature, extremely low birth weight and extremely low gestational age infants. BMC Ophthalmol..

[73] Dammann O., Hartnett M.E., Stahl A. Retinopathy of prematurity. Dev. Med. Child Neurol..

[74] Hellström A., Smith L.E., Dammann O. (2013). Retinopathy of prematurity. Lancet.

[75] Graziosi A., Perrotta M., Russo D., Gasparroni G., D’Egidio C., Marinelli B., Di Marzio G., Falconio G., Mastropasqua L., Volti G.L. (2020). Oxidative Stress Markers and the Retinopathy of Prematurity. J. Clin. Med..

[76] Stone W.L., Shah D., Hollinger S.M. (2016). Retinopathy of prematurity an oxidative stress neonatal disease. Front. Biosci..

[77] Saito Y., Geisen P., Uppal A., Hartnett M.E. (2007). Inhibition of NAD(P)H oxidase reduces apoptosis and avascular retina in an animal model of retinopathy of prematurity. Mol. Vis..

[78] Uno K., Prow T.W., Bhutto I.A., Yerrapureddy A., McLeod D.S., Yamamoto M., Reddy S.P., Lutty G.A. (2010). Role of Nrf2 in retinal vascular development and the vaso-obliterative phase of oxygen-induced retinopathy. Exp. Eye Res..

[79] Hartnett M.E., Penn J.S. (2012). Mechanisms and Management of Retinopathy of Prematurity. N. Engl. J. Med..

[80] Hartnett M.E. (2015). Pathophysiology and Mechanisms of Severe Retinopathy of Prematurity. Ophthalmology.

[81] Bonello S., Zähringer C., BelAiba R.S., Djordjevic T., Hess J., Michiels C., Kietzmann T., Görlach A. (2007). Reactive Oxygen Species Activate the HIF-1α Promoter Via a Functional NFκB Site. Arterioscler. Thromb. Vasc. Biol..

[82] Fevereiro-Martins M.D.R., Marques-Neves C.A.M., Guimarães H., Bicho M.D.P. (2023). Retinopathy of prematurity: A review of pathophysiology and signaling pathways. Surv. Ophthalmol..

[83] Beauchamp M.H., Martinez-Bermudez A.K., Gobeil F., Marrache A.M., Hou X., Speranza G., Abran D., Quiniou C., Lachapelle P., Roberts J. (2001). Role of thromboxane in retinal microvascular degeneration in oxygen-induced retinopathy. J. Appl. Physiol..

[84] Hou X., Gobeil F., Peri K., Speranza G., Marrache A.M., Lachapelle P., Roberts J., Varma D.R., Chemtob S. (2000). Augmented Vasoconstriction and Thromboxane Formation by 15-F 2t -Isoprostane (8-Iso-Prostaglandin F 2α ) in Immature Pig Periventricular Brain Microvessels. Stroke.

[85] Coleman R.J., Beharry K.D., Brock R.S., Abad-Santos P., Abad-Santos M., Modanlou H.D. (2008). Effects of Brief, Clustered Versus Dispersed Hypoxic Episodes on Systemic and Ocular Growth Factors in a Rat Model of Oxygen-Induced Retinopathy. Pediatr. Res..

[86] McColm J.R., Geisen P., Hartnett M.E. (2004). VEGF isoforms and their expression after a single episode of hypoxia or repeated fluctuations between hyperoxia and hypoxia: Relevance to clinical ROP. Mol. Vis..

[87] Di Fiore J.M., Kaffashi F., Loparo K., Sattar A., Schluchter M., Foglyano R., Martin R.J., Wilson C.G. (2012). The relationship between patterns of intermittent hypoxia and retinopathy of prematurity in preterm infants. Pediatr. Res..

[88] Di Fiore J.M., Bloom J.N., Orge F., Schutt A., Schluchter M., Cheruvu V.K., Walsh M., Finer N., Martin R.J. (2010). A Higher Incidence of Intermittent Hypoxemic Episodes Is Associated with Severe Retinopathy of Prematurity. J. Pediatr..

[89] Aranda J.V., Cai C.L., Ahmad T., Bronshtein V., Sadeh J., Valencia G.B., Lazzaro D.R., Beharry K.D. (2016). Pharmacologic synergism of ocular ketorolac and systemic caffeine citrate in rat oxygen-induced retinopathy. Pediatr. Res..

[90] Panfoli I., Candiano G., Malova M., De Angelis L.C., Cardiello V., Buonocore G., Ramenghi L.A. (2018). Oxidative Stress as a Primary Risk Factor for Brain Damage in Preterm Newborns. Front. Pediatr..

[91] Khwaja O., Volpe J.J. (2008). Pathogenesis of cerebral white matter injury of prematurity. Arch. Dis. Child. Fetal Neonatal Ed..

[92] French H.M., Reid M., Mamontov P., Simmons R.A., Grinspan J.B. (2009). Oxidative stress disrupts oligodendrocyte maturation. J. Neurosci. Res..

[93] Volpe J.J. (2009). Brain injury in premature infants: A complex amalgam of destructive and developmental disturbances. Lancet Neurol..

[94] Coviello C., Perrone S., Buonocore G., Negro S., Longini M., Dani C., de Vries L.S., Groenendaal F., Vijlbrief D.C., Benders M.J.N.L. (2021). Isoprostanes as Biomarker for White Matter Injury in Extremely Preterm Infants. Front. Pediatr..

[95] Ophelders D.R.M.G., Gussenhoven R., Klein L., Jellema R.K., Westerlaken R.J., Hütten M.C., Vermeulen J., Wassink G., Gunn A.J., Wolfs T.G. (2020). Preterm brain injury, antenatal triggers, and therapeutics: Timing is key. Cells.

[96] Brekke E., Berger H.R., Widerøe M., Sonnewald U., Morken T.S. (2017). Glucose and Intermediary Metabolism and Astrocyte–Neuron Interactions Following Neonatal Hypoxia–Ischemia in Rat. Neurochem. Res..

[97] Hope P.L., Cady E.B., Delpy D.T., Ives N.K., Gardiner R.M., Reynolds E.O.R. (1988). Brain Metabolism and Intracellular pH During Ischaemia: Effects of Systemic Glucose and Bicarbonate Administration Studied by31P and1H Nuclear Magnetic Resonance Spectroscopy In Vivo in the Lamb. J. Neurochem..

[98] Chung H.Y., Baek B.S., Song S.H., Kim M.S., Huh J.I., Shim K.H., Kim K.W., Lee K.H. (1997). Xanthine dehydrogenase/xanthine oxidase and oxidative stress. Age.

[99] Hagberg H., Mallard C., Rousset C.I., Thornton C. (2014). Mitochondria: Hub of injury responses in the developing brain. Lancet Neurol..

[100] Laptook A.R. (2016). Birth Asphyxia and Hypoxic-Ischemic Brain Injury in the Preterm Infant. Clin. Perinatol..

[101] Di Fiore J.M., Vento M. (2019). Intermittent hypoxemia and oxidative stress in preterm infants. Respir. Physiol. Neurobiol..

[102] Di Fiore J.M., Raffay T.M. (2021). The relationship between intermittent hypoxemia events and neural outcomes in neonates. Exp. Neurol..

[103] Back S.A. (2017). White matter injury in the preterm infant: Pathology and mechanisms. Acta Neuropathol..

[104] Liu S., Zhang X., Liu Y., Yuan X., Yang L., Zhang R., Zhang X., Wang X., Xu F., Zhu C. (2020). Early application of caffeine improves white matter development in very preterm infants. Respir. Physiol. Neurobiol..

[105] Reeder B.J. (2010). The Redox Activity of Hemoglobins: From Physiologic Functions to Pathologic Mechanisms. Antioxid. Redox Signal..

[106] Pandya C.D., Vekaria H., Joseph B., Slone S.A., Gensel J.C., Sullivan P.G., Miller B.A. (2021). Hemoglobin induces oxidative stress and mitochondrial dysfunction in oligodendrocyte progenitor cells. Transl. Res..

[107] Ballabh P., de Vries L.S. (2021). White matter injury in infants with intraventricular haemorrhage: Mechanisms and therapies. Nat. Rev. Neurol..

[108] Wan J., Ren H., Wang J. (2019). Iron toxicity, lipid peroxidation and ferroptosis after intracerebral haemorrhage. Stroke Vasc. Neurol..

[109] Romantsik O., Bruschettini M., Ley D. (2019). Intraventricular Hemorrhage and White Matter Injury in Preclinical and Clinical Studies. Neoreviews.

[110] Adler I., Batton D., Betz B., Bezinque S., Ecklund K., Junewick J., McCauley R., Miller C., Seibert J., Specter B. (2010). Mechanisms of injury to white matter adjacent to a large intraventricular hemorrhage in the preterm brain. J. Clin. Ultrasound.

[111] Zia M.T., Csiszar A., Labinskyy N., Hu F., Vinukonda G., LaGamma E.F., Ungvari Z., Ballabh P., Kim Y.M., Guzik T.J. (2009). Oxidative-Nitrosative Stress in a Rabbit Pup Model of Germinal Matrix Hemorrhage: Role of NAD(P)H oxidase. Stroke.

[112] Goulding D.S., Vogel R.C., Gensel J.C., Morganti J.M., Stromberg A.J., Miller B.A. (2020). Acute brain inflammation, white matter oxidative stress, and myelin deficiency in a model of neonatal intraventricular hemorrhage. J. Neurosurg. Pediatr..

[113] Jaganjac M., Cipak A., Schaur R.J., Zarkovic N. (2016). Pathophysiology of neutrophil-mediated extracellular redox reactions. Front. Biosci..

[114] Lu H.-Y., Zhang Q., Wang Q.-X., Lu J.-Y. (2016). Contribution of Histologic Chorioamnionitis and Fetal Inflammatory Response Syndrome to Increased Risk of Brain Injury in Infants With Preterm Premature Rupture of Membranes. Pediatr. Neurol..

[115] Anblagan D., Pataky R., Evans M.J., Telford E.J., Serag A., Sparrow S., Piyasena C., Semple S.I., Wilkinson A.G., Bastin M.E. (2016). Association between preterm brain injury and exposure to chorioamnionitis during fetal life. Sci. Rep..

[116] Procianoy R.S., Silveira R.C. (2012). Association between high cytokine levels with white matter injury in preterm infants with sepsis. Pediatr. Crit. Care Med..

[117] Gagliardi L., Bellu’ R., Zanini R., Dammann O. (2009). Bronchopulmonary dysplasia and brain white matter damage in the preterm infant: A complex relationship. Paediatr. Périnat. Epidemiol..

[118] Folkerth R.D., Keefe R.J., Haynes R.L., Trachtenberg F.L., Volpe J.J., Kinney H.C. (2004). Interferon-γ Expression in Periventricular Leukomalacia in the Human Brain. Brain Pathol..

[119] Kadhim H., Tabarki B., Verellen G., De Prez C., Rona A.-M., Sebire G. (2001). Inflammatory cytokines in the pathogenesis of periventricular leukomalacia. Neurology.

[120] Yoon B.H., Romero R., Kim C.J., Koo J.N., Choe G., Syn H.C., Chi J.-G. (1997). High expression of tumor necrosis factor-α and interleukin-6 in periventricular leukomalacia. Am. J. Obstet. Gynecol..

[121] Haynes R.L., Folkerth R.D., Keefe R.J., Sung I., Swzeda L.I., Rosenberg P., Volpe J.J., Kinney H.C. (2003). Nitrosative and Oxidative Injury to Premyelinating Oligodendrocytes in Periventricular Leukomalacia. J. Neuropathol. Exp. Neurol..

[122] Neu J., Walker W.A. (2011). Necrotizing Enterocolitis. N. Engl. J. Med..

[123] Fitzgibbons S.C., Ching Y., Yu D., Carpenter J., Kenny M., Weldon C., Lillehei C., Valim C., Horbar J.D., Jaksic T. (2009). Mortality of necrotizing enterocolitis expressed by birth weight categories. J. Pediatr. Surg..

[124] Hackam D.J., Sodhi C.P. (2022). Bench to bedside—New insights into the pathogenesis of necrotizing enterocolitis. Nat. Rev. Gastroenterol. Hepatol..

[125] Aydemir C., Dilli D., Uras N., Ulu H.O., Oguz S.S., Erdeve O., Dilmen U. (2011). Total oxidant status and oxidative stress are increased in infants with necrotizing enterocolitis. J. Pediatr. Surg..

[126] Perrone S., Tataranno M.L., Negro S., Cornacchione S., Longini M., Proietti F., Soubasi V., Benders M.J., Van Bel F., Buonocore G. (2012). May oxidative stress biomarkers in cord blood predict the occurrence of necrotizing enterocolitis in preterm infants?. J. Matern. Fetal Neonatal Med..

[127] Yazji I., Sodhi C.P., Lee E.K., Good M., Egan C.E., Afrazi A., Neal M.D., Jia H., Lin J., Ma C. (2013). Endothelial TLR4 activation impairs intestinal microcirculatory perfusion in necrotizing enterocolitis via eNOS–NO–nitrite signaling. Proc. Natl. Acad. Sci. USA.

[128] Ferretti E., Tremblay E., Thibault M.-P., Grynspan D., Burghardt K.M., Bettolli M., Babakissa C., Levy E., Beaulieu J.-F. (2017). The nitric oxide synthase 2 pathway is targeted by both pro- and anti-inflammatory treatments in the immature human intestine. Nitric Oxide.

[129] Whitehouse J.S., Xu H., Shi Y., Noll L., Kaul S., Jones D.W., Pritchard K.A., Oldham K.T., Gourlay D.M. (2010). Mesenteric Nitric Oxide and Superoxide Production in Experimental Necrotizing Enterocolitis. J. Surg. Res..

[130] Grishin A., Bowling J., Bell B., Wang J., Ford H.R. (2016). Roles of nitric oxide and intestinal microbiota in the pathogenesis of necrotizing enterocolitis. J. Pediatr. Surg..

[131] Chen C.-A., Wang T.-Y., Varadharaj S., Reyes L.A., Hemann C., Hassan Talukder M.A., Chen Y.-R., Druhan L.J., Zweier J.L. (2010). S-glutathionylation uncouples eNOS and regulates its cellular and vascular function. Nature.

[132] Sullivan S., Schanler R.J., Kim J.H., Patel A.L., Trawöger R., Kiechl-Kohlendorfer U., Chan G.M., Blanco C.L., Abrams S., Cotten C.M. (2010). An Exclusively Human Milk-Based Diet Is Associated with a Lower Rate of Necrotizing Enterocolitis than a Diet of Human Milk and Bovine Milk-Based Products. J. Pediatr..

[133] Shoji H., Shimizu T., Shinohara K., Oguchi S., Shiga S., Yamashiro Y. (2004). Suppressive effects of breast milk on oxidative DNA damage in very low birthweight infants. Arch. Dis. Child. Fetal Neonatal Ed..

[134] Friel J.K., Martin S.M., Langdon M., Herzberg G.R., Buettner G.R. (2002). Milk from Mothers of Both Premature and Full-Term Infants Provides Better Antioxidant Protection than Does Infant Formula. Pediatr. Res..

[135] Friel J.K., Diehl-Jones B., Cockell K.A., Chiu A., Rabanni R., Davies S.S., Roberts L.J. (2011). Evidence of Oxidative Stress in Relation to Feeding Type During Early Life in Premature Infants. Pediatr. Res..

[136] Good M., Sodhi C.P., Yamaguchi Y., Jia H., Lu P., Fulton W.B., Martin L.Y., Prindle T., Nino D.F., Zhou Q. (2016). The human milk oligosaccharide 2′-fucosyllactose attenuates the severity of experimental necrotising enterocolitis by enhancing mesenteric perfusion in the neonatal intestine. Br. J. Nutr..

[137] Kim M., Christley S., Alverdy J.C., Liu D., An G. (2012). Immature Oxidative Stress Management as a Unifying Principle in the Pathogenesis of Necrotizing Enterocolitis: Insights from an Agent-Based Model. Surg. Infect..

[138] Carlisle E.M., Morowitz M.J. (2013). The intestinal microbiome and necrotizing enterocolitis. Curr. Opin. Pediatr..

[139] Stewart C.J., Embleton N.D., Marrs E.C.L., Smith D.P., Nelson A., Abdulkadir B., Skeath T., Petrosino J.F., Perry J.D., Berrington J.E. (2016). Temporal bacterial and metabolic development of the preterm gut reveals specific signatures in health and disease. Microbiome.

[140] Pammi M., Cope J., Tarr P.I., Warner B.B., Morrow A.L., Mai V., Gregory K.E., Kroll J.S., McMurtry V., Ferris M.J. (2017). Intestinal dysbiosis in preterm infants preceding necrotizing enterocolitis: A systematic review and meta-analysis. Microbiome.

[141] Cai C., Zhang Z., Morales M., Wang Y., Khafipour E., Friel J. (2019). Feeding practice influences gut microbiome composition in very low birth weight preterm infants and the association with oxidative stress: A prospective cohort study. Free. Radic. Biol. Med..

[142] Li X., Li X., Shang Q., Gao Z., Hao F., Guo H., Guo C. (2017). Fecal microbiota transplantation (FMT) could reverse the severity of experimental necrotizing enterocolitis (NEC) via oxidative stress modulation. Free. Radic. Biol. Med..

[143] Prado C., Michels M., Ávila P., Burger H., Milioli M.V.M., Dal-Pizzol F. (2019). The protective effects of fecal microbiota transplantation in an experimental model of necrotizing enterocolitis. J. Pediatr. Surg..

[144] Goldstein S.L., Somers M.J., Baum M.A., Symons J.M., Brophy P.D., Blowey D., Bunchman T.E., Baker C., Mottes T., Mcafee N. (2005). Pediatric patients with multi-organ dysfunction syndrome receiving continuous renal replacement therapy. Kidney Int..

[145] Stoll B.J., Hansen N.I., Bell E.F., Shankaran S., Laptook A.R., Walsh M.C., Hale E.C., Newman N.S., Schibler K., Carlo W.A. (2010). Neonatal Outcomes of Extremely Preterm Infants from the NICHD Neonatal Research Network. Pediatrics.

[146] Spasojević I., Obradović B., Spasić S. (2012). Bench-to-bedside review: Neonatal sepsis—Redox processes in pathogenesis. Crit. Care.

[147] Volante E., Moretti S., Pisani F., Bevilacqua G. (2004). Early diagnosis of bacterial infection in the neonate. J. Matern. Fetal Neonatal Med..

[148] Segura-Cervantes E., Mancilla-Ramírez J., González-Canudas J., Alba E., Santillán-Ballesteros R., Morales-Barquet D., Sandoval-Plata G., Galindo-Sevilla N. (2016). Inflammatory Response in Preterm and Very Preterm Newborns with Sepsis. Mediat. Inflamm..

[149] Poggi C., Dani C. (2018). Sepsis and Oxidative Stress in the Newborn: From Pathogenesis to Novel Therapeutic Targets. Oxidative Med. Cell. Longev..

[150] Figueras-Aloy J., Gómez L., Rodríguez-Miguélez J.M., Jordán Y., Salvia M.D., Jiménez W., Carbonell-Estrany X. (2003). Plasma nitrite/nitrate and endothelin-1 concentrations in neonatal sepsis. Acta Paediatr..

[151] Marom D., Yuhas Y., Sirota L., Livni G., Ashkenazi S. (2004). Nitric Oxide Levels in Preterm and Term Infants and in Premature Infants with Bacteremia. Biol. Neonate.

[152] Mittal R., Gonzalez-Gomez I., Goth K.A., Prasadarao N.V. (2010). Inhibition of Inducible Nitric Oxide Controls Pathogen Load and Brain Damage by Enhancing Phagocytosis of Escherichia coli K1 in Neonatal Meningitis. Am. J. Pathol..

[153] Özalkaya E., Karatekin G., Topçuoğlu S., Karatepe H., Hafızoğlu T., Baran P., Ovalı F. (2017). Neonatology oxidative status in preterm infants with premature preterm rupture of membranes and fetal inflammatuar response syndrome. Pediatr. Neonatol..

[154] Bharadwaj S.K., Bhat B.V., Vickneswaran V., Adhisivam B., Bobby Z., Habeebullah S. (2018). Oxidative Stress, Antioxidant Status and Neurodevelopmental Outcome in Neonates Born to Pre-eclamptic Mothers. Indian J. Pediatr..

[155] Coutinho F.G., Diniz E.M.D.A., Kandler I., Cianciarullo M.A., Dos Santos N.R. (2018). Assessment of oxidative damage and enzymatic antioxidant system activity on the umbilical cord blood and saliva from preterm newborns with risk factors for early-onset neonatal sepsis. Rev. Assoc. Med. Bras..

[156] Cancelier A.C., Petronilho F., Reinke A., Constantino L., Machado R., Ritter C., Dal-Pizzol F. (2009). Inflammatory and oxidative parameters in cord blood as diagnostic of early-onset neonatal sepsis: A case-control study. Pediatr. Crit. Care Med..

[157] Asci A., Surmeli-Onay O., Erkekoglu P., Yigit S., Yurdakok M., Kocer-Gumusel B. (2015). Oxidant and antioxidant status in neonatal proven and clinical sepsis according to selenium status. Pediatr. Int..

[158] Gitto E., Karbownik M., Reiter R.J., Tan D.X., Cuzzocrea S., Chiurazzi P., Cordaro S., Corona G., Trimarchi G., Barberi I. (2001). Effects of Melatonin Treatment in Septic Newborns. Pediatr. Res..

[159] Batra S., Kumar R., Kapoor A.K., Ray G. (2000). Alterations in antioxidant status during neonatal sepsis. Ann. Trop. Paediatr..

[160] Valerio T.A., Cancelier A.C., Constantino L., Petronilho F., Ritter C., Dal-Pizzol F. (2012). Inflammatory and oxidative cord blood parameters as predictors of neonatal sepsis severity. Rev. Bras. Ter. Intensiv..

[161] Kapoor K., Basu S., Das B.K., Bhatia B.D. (2006). Lipid Peroxidation and Antioxidants in Neonatal Septicemia. J. Trop. Pediatr..

[162] Khan N.A., Singla M., Samal S., Lodha R., Medigeshi G.R. (2020). Respiratory Syncytial Virus-Induced Oxidative Stress Leads to an Increase in Labile Zinc Pools in Lung Epithelial Cells. Msphere.

[163] Kumova O.K., Galani I.-E., Rao A., Johnson H., Triantafyllia V., Matt S.M., Pascasio J., Gaskill P.J., Andreakos E., Katsikis P.D. (2022). Severity of neonatal influenza infection is driven by type I interferon and oxidative stress. Mucosal Immunol..

